# Independent factors associated with renal impairment in preeclampsia and its association with maternal and neonatal adverse outcomes

**DOI:** 10.3389/fmed.2026.1832755

**Published:** 2026-07-08

**Authors:** Zi-Xin Tao, Ai-Yue Chen, Yu Yang, Na Yang, Zhen Zeng, Yan-Ling Wu, Bei-Lan Zeng, Yu-Jie Zhang, Yang Cheng

**Affiliations:** Department of Obstetrics and Gynecology, Guangzhou First People’s Hospital, South China University of Technology, Guangzhou, China

**Keywords:** acute kidney injury, maternal outcomes, mean arterial pressure, preeclampsia, renal impairment

## Abstract

**Background:**

Renal impairment is a clinically important manifestation of preeclampsia and may identify patients at increased risk of adverse maternal and neonatal outcomes. This study evaluated independent factors associated with renal impairment in preeclampsia and examined the association between renal impairment and in-hospital maternal and neonatal adverse outcomes.

**Methods:**

This retrospective cohort study included 333 women with preeclampsia (PE) managed between June 2021 and June 2025. Patients were classified into renal-impairment and control groups. Baseline characteristics, laboratory variables, treatment exposure, and in-hospital outcomes were compared using appropriate parametric, nonparametric, or categorical tests. Univariable and multivariable logistic regression analyses were performed to identify factors associated with renal impairment and to evaluate its association with adverse outcomes. Model calibration, discrimination, and multicollinearity were assessed using the Hosmer–Lemeshow test, receiver operating characteristic curve analysis, and variance inflation factors, respectively. Sensitivity and subgroup analyses were performed according to alternative renal-impairment definitions and PE onset type.

**Results:**

In the primary multivariable model, higher body mass index (BMI), mean arterial pressure (MAP), and uric acid were independently associated with renal impairment, whereas later gestational age, higher albumin, and higher platelet count were inversely associated. In the expanded model, early-onset PE (adjusted odds ratio [aOR] 1.68, 95% confidence interval [CI] 1.02–2.78), MAP (aOR 1.73, 95% CI 1.26–2.37), 24-h urinary protein (aOR 1.58, 95% CI 1.25–2.00), serum uric acid-to-creatinine ratio (aOR 0.36, 95% CI 0.26–0.51), and albumin (aOR 0.87, 95% CI 0.81–0.94) remained associated with renal impairment. Renal impairment was independently associated with composite adverse maternal outcome (aOR 2.08, 95% CI 1.20–3.59), intensive care unit admission (aOR 3.39, 95% CI 1.65–6.95), eclampsia (aOR 3.92, 95% CI 1.09–14.14), preterm birth, low birth weight, low 5-min Apgar score, and neonatal intensive care unit admission.

**Conclusion:**

In preeclampsia, renal impairment was independently associated with hemodynamic severity, biochemical markers, and increased odds of major maternal and neonatal adverse outcomes. These findings suggest that renal impairment might serve as a clinically relevant marker for in-hospital risk stratification, although prospective validation is needed.

## 1 Introduction

Hypertensive disorders of pregnancy, particularly preeclampsia, remain major causes of maternal morbidity and perinatal complications worldwide (1). Contemporary cardiovascular guidance emphasizes that prompt recognition and management of pregnancy-related hypertension are essential to reduce end-organ injury, including acute kidney injury (AKI), and to mitigate subsequent maternal and fetal risk (2, 3). Renal involvement is a clinically significant manifestation of preeclampsia, reflecting systemic endothelial dysfunction and microvascular injury that may progress rapidly and require intensified monitoring or critical care (4). Recent nephrology consensus statements have identified pregnancy-associated AKI as a priority condition for standardized evaluation and risk-stratified care pathways because of its acute severity and potential for persistent renal sequelae (5, 6). The implications of renal impairment in preeclampsia extend beyond the index hospitalization (7). Population-based and longitudinal studies have shown that preeclampsia is associated with an increased risk of subsequent maternal kidney disease and may also carry intergenerational implications for offspring renal health (8). These findings underscore the importance of identifying patients at increased risk of renal impairment during the index pregnancy, not only to optimize immediate clinical management but also to inform postpartum surveillance and follow-up strategies (9).

Risk stratification is increasingly supported by both conventional and emerging biomarkers. Angiogenic imbalance, commonly reflected by circulating soluble fms-like tyrosine kinase-1 (sFlt-1) and placental growth factor (PlGF), has demonstrated diagnostic and prognostic value in preeclampsia, including among high-risk populations, and is associated with adverse maternal and perinatal outcomes (10, 11). In parallel, readily available clinical indices, including blood pressure severity measures and routine laboratory parameters, remain clinically actionable tools for early risk identification. The 2024 European position statement on hypertensive disorders in pregnancy explicitly incorporates renal dysfunction thresholds into the spectrum of maternal organ dysfunction, further underscoring the central role of kidney assessment in disease classification and management (12). Among routine laboratory markers, uric acid, serum albumin, and platelet count are frequently regarded as indicators of disease severity and endothelial injury (13). Recent observational studies suggest that combinations of blood pressure measures and biochemical indicators, including uric acid and albuminuria-related indices, may improve prediction of severe preeclampsia phenotypes. In addition, low serum albumin has been evaluated as a marker of preeclampsia severity with relevance to maternal and fetal complications, consistent with a phenotype characterized by capillary leak and protein loss (14, 15). Inflammatory activation may further influence renal susceptibility, and recent analyses have examined systemic inflammatory indices in relation to preeclampsia-associated AKI (16).

Given the clinical consequences of renal impairment for pregnancy trajectories, it is also important to consider renal dysfunction in the context of perinatal outcomes (17, 18). Maternal systemic illness contributes to prematurity and neonatal morbidity, and neonatal AKI has been associated with adverse outcomes in extremely preterm infants, further highlighting the potential downstream effects of pregnancy complications that lead to early delivery (19). Against this background, the present study aimed to identify clinical and laboratory factors associated with renal impairment and to examine whether renal impairment was associated with in-hospital maternal and neonatal adverse outcomes, with the goal of supporting objective, risk-oriented clinical decision-making in patients with hypertensive disorders of pregnancy.

## 2 Methods

### 2.1 Study design

This retrospective cohort study consecutively enrolled patients diagnosed with preeclampsia who received inpatient management at our institution between June 2021 and June 2025. Participants were stratified according to renal involvement: the exposure (renal-impairment) group comprised women with preeclampsia complicated by renal impairment, whereas the control group included women with preeclampsia without renal impairment and without evidence of other major end-organ injury. Eligible patients were those with a confirmed diagnosis of preeclampsia based on contemporaneous clinical criteria and with complete medical records sufficient for ascertainment of renal function indices and maternal–neonatal outcomes. Exclusion criteria were: (1) pre-existing chronic kidney disease or known structural renal disease prior to pregnancy; (2) chronic hypertension predating pregnancy; (3) pregestational diabetes mellitus; (4) known systemic autoimmune disease (systemic lupus erythematosus or antiphospholipid syndrome); (5) acute or chronic liver disease or other primary hepatic disorders; (6) active urinary tract infection or other conditions likely to confound renal biomarkers; (7) multiple gestation; (8) major fetal congenital anomalies; (9) missing key laboratory variables required to define renal impairment or incomplete outcome data; and (10) transfer to another facility before delivery or definitive outcome assessment. The study protocol was reviewed and approved by the institutional ethics committee of our hospital. Informed consent was obtained from all participants. All procedures were conducted in accordance with the Declaration of Helsinki and relevant guidelines and regulations. All data were anonymized prior to analysis to ensure participant confidentiality.

### 2.2 Diagnostic criteria

Preeclampsia diagnosis. Preeclampsia was diagnosed in accordance with the American College of Obstetricians and Gynecologists (ACOG) Practice Bulletin No. 222 (2020) criteria. Briefly, preeclampsia was defined as new-onset hypertension after 20 weeks of gestation accompanied by proteinuria (24-h urinary protein ≥300 mg or urine protein-to-creatinine ratio [UPCR] ≥ 0.30) or, in the absence of proteinuria, maternal end-organ dysfunction (20, 21).

Operational definition of renal impairment (grouping definition). Renal impairment was defined using objective renal function criteria. Patients were classified as having renal impairment if serum creatinine exceeded 1.1 mg/dL (≈97 μmol/L) or doubled from a documented baseline, and/or if AKI criteria were met (KDIGO-based serum creatinine change and/or oliguria when urine output data were available). Renal status was determined using laboratory results obtained at admission and the worst value recorded prior to delivery or discharge (21).

Definition of other end-organ injury (for control-group exclusion). To minimize baseline confounding, the control group included patients without renal impairment and without other major end-organ injury, defined as any of the following: hepatic involvement (transaminases ≥2 × the upper limit of normal and/or persistent right upper quadrant/epigastric pain attributable to liver involvement), hematologic involvement (platelet count <100 × 10^9^/L), neurologic symptoms (new-onset severe headache unresponsive to treatment and/or visual disturbances), or pulmonary edema confirmed clinically and/or radiographically (21).

### 2.3 Data collection and measurements

Baseline demographics and obstetric characteristics. Maternal demographic and pregnancy-related variables were extracted from the electronic medical record, including age at admission, body mass index (BMI), gestational age at preeclampsia diagnosis, gravidity and parity, and a history of adverse obstetric outcomes (e.g., prior preeclampsia, preterm birth, fetal growth restriction, or stillbirth, when documented). Preeclampsia onset type was classified according to gestational age at diagnosis: early-onset preeclampsia was defined as diagnosis before 34 weeks of gestation, whereas late-onset preeclampsia was defined as diagnosis at or after 34 weeks of gestation.

Clinical manifestations, vital signs, and treatment exposure. Blood pressure measurements at the time of diagnosis were recorded, including systolic blood pressure (SBP), diastolic blood pressure (DBP), and mean arterial pressure (MAP, calculated as DBP + 1/3[SBP − DBP]). Key symptoms and signs at presentation were captured, such as headache and visual disturbances. Treatment-related variables during the index hospitalization were documented, including magnesium sulfate prophylaxis/therapy and antihypertensive management (drug class and/or regimen).

Laboratory variables and ancillary examinations. Laboratory indices were collected using samples obtained on the day of preeclampsia diagnosis. Renal-related markers included serum creatinine (SCr), blood urea nitrogen (BUN), uric acid, estimated glomerular filtration rate (eGFR, calculated using a prespecified equation), urine protein assessment (urine protein-to-creatinine ratio [UPCR] and/or 24-h urinary protein, if performed), and routine urinalysis. The serum uric acid-to-creatinine ratio was calculated as serum uric acid divided by serum creatinine, with both variables expressed in μmol/L. Hematologic parameters included hemoglobin, platelet count, and coagulation profiles. Hepatic injury markers included aspartate aminotransferase (AST), alanine aminotransferase (ALT), lactate dehydrogenase (LDH), and total bilirubin. Inflammatory and metabolic variables included serum albumin and C-reactive protein (CRP). Results of ancillary examinations (e.g., ultrasound or other clinically indicated assessments) were recorded when performed as part of routine care.

Data extraction was performed using a standardized case report form. Two investigators independently entered and cross-checked the dataset, with discrepancies resolved by review of the source records. Implausible or out-of-range values were verified against the original laboratory reports and clinical documentation; where transcription errors were identified, corrections were made based on the primary source.

### 2.4 Maternal and neonatal outcome definitions

Maternal and neonatal outcomes were ascertained from the electronic medical record and were evaluated during the index hospitalization. Adverse maternal outcomes included intensive care unit (ICU) admission, eclampsia, HELLP syndrome (hemolysis, elevated liver enzymes, and low platelet count), postpartum hemorrhage, disseminated intravascular coagulation (DIC), acute heart failure and/or pulmonary edema, and placental abruption. Adverse neonatal outcomes included preterm birth, low birth weight, low Apgar score, and neonatal ICU (NICU) admission. All outcomes were defined *a priori* and were adjudicated based on clinician documentation and contemporaneous laboratory and/or imaging findings when applicable.

### 2.5 Statistical analysis

All statistical analyses were performed using IBM SPSS Statistics, version 28.0 (IBM Corp., Armonk, NY, USA) and R software, version 4.3.2 (R Foundation for Statistical Computing, Vienna, Austria). Continuous variables were summarized as mean ± standard deviation or median with interquartile range, and categorical variables as counts and percentages. Between-group comparisons were performed using the independent-samples t test, Mann–Whitney U test, chi-square test, or Fisher’s exact test, as appropriate. Univariable logistic regression was used to estimate crude odds ratios (ORs) with 95% confidence intervals (CIs). Variables with a univariable *p* value <0.10 and clinically relevant covariates were considered for multivariable logistic regression. The primary model was fitted using the enter method, without automated forward, backward, or stepwise selection. Model calibration, discrimination, and multicollinearity were assessed using the Hosmer–Lemeshow test, receiver operating characteristic curve analysis, and variance inflation factors, respectively. An expanded clinically adjusted model was constructed to incorporate PE onset type, 24-h urinary protein, and serum uric acid-to-creatinine ratio. Stratified and reduced multivariable models were further performed according to early-onset and late-onset PE. Sensitivity analyses evaluated alternative renal-impairment definitions, and subgroup analyses assessed interaction effects. Two-sided *p* < 0.05 was considered statistically significant.

## 3 Results

### 3.1 Study population and patient selection

During the study period (June 2021 to June 2025), a total of 389 consecutive inpatients with a diagnosis of preeclampsia were screened for eligibility. After applying the predefined exclusion criteria, 56 patients were excluded, including pre-existing chronic kidney disease or known structural renal disease (*n* = 9), chronic hypertension predating pregnancy (*n* = 12), pregestational diabetes mellitus (*n* = 6), systemic autoimmune disease (systemic lupus erythematosus and/or antiphospholipid syndrome) (*n* = 5), acute or chronic liver disease or other primary hepatic disorders (*n* = 4), active urinary tract infection or other conditions likely to confound renal biomarkers (*n* = 7), multiple gestation (*n* = 5), major fetal congenital anomalies (*n* = 2), missing key laboratory variables required to define renal impairment and/or incomplete outcome data (*n* = 4), and transfer to another facility before delivery or definitive outcome assessment (*n* = 2). Consequently, 333 patients were included in the final analysis. Of these, 128 were assigned to the renal-impairment group, and 205 to the control group (preeclampsia without renal impairment and without other major end-organ injury).

### 3.2 Baseline characteristics and clinical profile at diagnosis

Compared with controls, patients in the renal-impairment group had a higher BMI (28.09 ± 3.86 vs. 26.57 ± 3.82 kg/m^2^, *p* = 0.001) and were diagnosed at an earlier gestational age (31.72 ± 3.56 vs. 32.91 ± 3.44 weeks, *p* = 0.003). Consistently, the distribution of PE onset type differed significantly between groups (*p* = 0.011), with a higher proportion of early-onset PE in the renal-impairment group than in the control group (75.0% vs. 61.5%). At diagnosis, the renal-impairment group exhibited higher blood pressure levels (SBP, DBP, and MAP; all *p* ≤ 0.026). Regarding obstetric history, prior preterm birth and prior FGR/SGA were more frequent in the renal-impairment group (*p* = 0.015 and *p* = 0.002, respectively), whereas prior preeclampsia and stillbirth history did not differ significantly. Visual disturbance at presentation was more common in the renal-impairment group (18.0% vs. 9.3%, *p* = 0.020), and antihypertensive therapy was used more frequently (93.8% vs. 81.5%, *p* = 0.002). Aspirin use during pregnancy was uncommon and did not differ significantly between groups (2.3% vs. 3.9%, *p* = 0.541; Table 1).

**Table 1:** Baseline demographic, obstetric, clinical, and treatment characteristics by renal impairment status.

Variable	Renal impairment (*n* = 128)	Control (*n* = 205)	Test statistic	*p* value
Age, years	30.97 ± 4.63	30.67 ± 6.03	*t* = 0.52	0.604
BMI, kg/m^2^	28.09 ± 3.86	26.57 ± 3.82	*t* = 3.51	0.001*
Gestational age at PE diagnosis, weeks	31.72 ± 3.56	32.91 ± 3.44	*t* = −3.01	0.003*
SBP at diagnosis, mmHg	164.13 ± 13.92	159.38 ± 15.27	*t* = 2.92	0.004*
DBP at diagnosis, mmHg	103.99 ± 11.11	101.28 ± 10.17	*t* = 2.24	0.026*
MAP at diagnosis, mmHg	124.04 ± 8.43	120.64 ± 8.29	*t* = 3.60	<0.001*
Gravidity, median [IQR]	3 [2–4]	3 [2–4]	U = 13,763	0.441
Parity, median [IQR]	1 [0–2]	1 [0–2]	U = 12,739	0.637
History of preeclampsia, *n* (%)	25 (19.5)	27 (13.2)	*χ*^2^ = 2.42	0.120
History of preterm birth, *n* (%)	26 (20.3)	22 (10.7)	*χ*^2^ = 5.86	0.015*
History of FGR/SGA, *n* (%)	22 (17.2)	13 (6.3)	*χ*^2^ = 9.86	0.002*
History of stillbirth, *n* (%)	6 (4.7)	7 (3.4)	*χ*^2^ = 0.34	0.560
Headache at presentation, *n* (%)	44 (34.4)	54 (26.3)	*χ*^2^ = 2.45	0.118
Visual disturbance, *n* (%)	23 (18.0)	19 (9.3)	*χ*^2^ = 5.41	0.020*
Magnesium sulfate use, *n* (%)	109 (85.2)	159 (77.6)	*χ*^2^ = 2.89	0.089
Antihypertensive therapy, *n* (%)	120 (93.8)	167 (81.5)	*χ*^2^ = 9.99	0.002*
PE onset type, *n* (%)			*χ*^2^ = 6.50	0.011*
Early-onset PE	96 (75.0)	126 (61.5)		
Late-onset PE	32 (25.0)	79 (38.5)		
Aspirin use during pregnancy, *n* (%)	3 (2.3)	8 (3.9)	Fisher’s exact test	0.541

Data are presented as mean ± SD, median [IQR], or n (%) as appropriate. *p < 0.05. Early-onset PE was defined as PE diagnosed before 34 weeks of gestation, whereas late-onset PE was defined as PE diagnosed at or after 34 weeks of gestation. The *χ*^2^ statistic and p value refer to the overall comparison of PE onset type between groups. BMI, body mass index; DBP, diastolic blood pressure; FGR, fetal growth restriction; IQR, interquartile range; MAP, mean arterial pressure; PE, preeclampsia; SBP, systolic blood pressure; SD, standard deviation; SGA, small for gestational age.

### 3.3 Laboratory and ancillary examination findings at the time of preeclampsia diagnosis

On the day of preeclampsia diagnosis, the renal-impairment group exhibited a laboratory profile showing poorer renal function, including higher SCr (114.18 ± 19.41 vs. 71.01 ± 11.24 μmol/L), higher BUN (6.67 ± 1.60 vs. 4.93 ± 1.24 mmol/L), higher uric acid (417.63 ± 75.90 vs. 362.83 ± 64.88 μmol/L), and lower eGFR (76.50 ± 18.57 vs. 104.67 ± 16.19 mL/min/1.73 m^2^), all *p* < 0.001. Proteinuria burden was also greater in the renal-impairment group, as reflected by higher UPCR and 24-h urinary protein (both p < 0.001) and a shift toward higher dipstick protein grades (*p* = 0.003), whereas hematuria frequency did not differ significantly (*p* = 0.258). Despite excluding patients with non-renal end-organ injury from the control group, modest between-group differences persisted in platelet count (*p* = 0.006) and PT (*p* = 0.033), and D-dimer was higher in the renal-impairment group (*p* = 0.003). Liver enzymes (AST/ALT) and LDH were not significantly different, although total bilirubin was slightly higher in the renal-impairment group (*p* < 0.001). Albumin was lower and CRP was higher in the renal-impairment group (both p < 0.001), which may reflect greater systemic inflammation and/or hemodilution at presentation (Table 2).

**Table 2:** Laboratory and urinalysis findings by renal impairment status.

Variable	Renal impairment (*n* = 128)	Control (*n* = 205)	Test statistic	*p* value
Serum creatinine (SCr), μmol/L	114.18 ± 19.41	71.01 ± 11.24	*t* = 22.88	<0.001*
Blood urea nitrogen (BUN), mmol/L	6.67 ± 1.60	4.93 ± 1.24	*t* = 10.52	<0.001*
Uric acid, μmol/L	417.63 ± 75.90	362.83 ± 64.88	*t* = 6.77	<0.001*
eGFR, mL/min/1.73 m^2^	76.50 ± 18.57	104.67 ± 16.19	*t* = −14.14	<0.001*
UPCR, g/g, median [IQR]	1.09 [0.76–1.70]	0.56 [0.41–0.84]	U = 21,279	<0.001*
24-h urinary protein, g/24 h, median [IQR]	2.02 [1.40–2.82]	1.01 [0.64–1.55]	U = 20,801	<0.001*
Urine protein (dipstick), *n* (%)†	12 (9.4) / 36 (28.1) / 48 (37.5) / 32 (25.0)	34 (16.6) / 83 (40.5) / 60 (29.3) / 28 (13.7)	*χ*^2^ = 13.61	0.003*
Hematuria (≥1+), *n* (%)	31 (24.2)	39 (19.0)	*χ*^2^ = 1.28	0.258
Hemoglobin, g/L	111.25 ± 14.11	113.26 ± 12.01	*t* = −1.33	0.184
Platelet count, ×10^9^/L	179.83 ± 42.59	193.31 ± 43.42	*t* = −2.79	0.006*
Prothrombin time (PT), s	12.62 ± 1.10	12.36 ± 1.01	*t* = 2.14	0.033*
Activated partial thromboplastin time (APTT), s	31.55 ± 4.27	31.13 ± 3.97	*t* = 0.89	0.376
Fibrinogen, g/L	4.25 ± 0.81	4.32 ± 0.81	*t* = −0.82	0.416
D-dimer, mg/L FEU, median [IQR]	1.57 [1.03–2.70]	1.31 [0.88–2.04]	U = 15,627	0.003*
AST, U/L	31.86 ± 12.76	30.01 ± 9.99	*t* = 1.40	0.163
ALT, U/L	27.98 ± 14.09	25.28 ± 12.46	*t* = 1.78	0.077
LDH, U/L	306.71 ± 91.33	289.91 ± 79.74	*t* = 1.71	0.088
Total bilirubin, μmol/L	11.18 ± 3.22	9.91 ± 3.15	*t* = 3.54	<0.001*
Albumin, g/L	33.34 ± 3.87	34.89 ± 4.09	*t* = −3.48	<0.001*
CRP, mg/L, median [IQR]	8.96 [5.30–15.35]	5.86 [3.79–9.04]	U = 16,870	<0.001*
Serum uric acid-to-creatinine ratio	3.76 ± 0.82	5.24 ± 1.05	*t* = −14.26	<0.001*

Data are presented as mean ± SD, median [IQR], or *n* (%) as appropriate. †Urine protein dipstick categories are presented as negative/trace / 1+ / 2+ / ≥3+, respectively. *p < 0.05. ALT, alanine aminotransferase; APTT, activated partial thromboplastin time; AST, aspartate aminotransferase; BUN, blood urea nitrogen; CRP, C-reactive protein; eGFR, estimated glomerular filtration rate; FEU, fibrinogen equivalent units; Hb, hemoglobin; IQR, interquartile range; LDH, lactate dehydrogenase; PT, prothrombin time; SCr, serum creatinine; SD, standard deviation; UPCR, urine protein-to-creatinine ratio.

### 3.4 Univariable associations with renal impairment

In univariable logistic regression analyses, higher BMI, higher blood pressure indices (SBP, DBP, and MAP), gravidity, the presence of headache and visual disturbance, higher uric acid levels, and higher CRP were each associated with increased odds of renal impairment (all *p* ≤ 0.045). Early-onset PE was also associated with higher odds of renal impairment compared with late-onset PE (OR 1.88, 95% CI 1.15–3.07; *p* = 0.011). In contrast, greater gestational age at diagnosis, higher serum albumin, and higher platelet count were inversely associated with renal impairment (all *p* < 0.001). Age and parity were not significantly associated with renal impairment in unadjusted models. These candidate variables were considered for inclusion in subsequent multivariable modeling to identify factors associated with renal impairment (Table 3).

**Table 3:** Univariable logistic regression analysis of candidate factors associated with renal impairment in preeclampsia.

Candidate variable	OR (95% CI)	*p* value
Age (per 1 year)	1.02 (0.98–1.06)	0.304
BMI (per 1 kg/m^2^)	1.11 (1.05–1.18)	<0.001*
Gestational age at diagnosis (per 1 week)	0.89 (0.83–0.95)	<0.001*
Gravidity (per 1)	1.18 (1.01–1.39)	0.040*
Parity (per 1)	1.07 (0.83–1.37)	0.615
SBP (per 10 mmHg)	1.17 (1.01–1.37)	0.038*
DBP (per 10 mmHg)	1.51 (1.21–1.90)	<0.001*
MAP (per 10 mmHg)	1.81 (1.36–2.40)	<0.001*
Headache (yes vs. no)	1.64 (1.01–2.65)	0.045*
Visual disturbance (yes vs. no)	3.41 (1.67–6.97)	<0.001*
Uric acid (per 50 μmol/L)	1.83 (1.52–2.21)	<0.001*
Albumin (per 1 g/L)	0.90 (0.85–0.96)	<0.001*
Platelet count (per 10 × 10^9^/L)	0.91 (0.86–0.96)	<0.001*
CRP (per 5 mg/L)	1.36 (1.17–1.59)	<0.001*
Early-onset PE, <34 weeks vs. ≥ 34 weeks	1.88 (1.15–3.07)	0.011*

*p < 0.05. BMI, body mass index; CI, confidence interval; CRP, C-reactive protein; DBP, diastolic blood pressure; MAP, mean arterial pressure; OR, odds ratio; SBP, systolic blood pressure.

### 3.5 Multivariable logistic regression for renal impairment

In the multivariable logistic regression model, covariates were entered *a priori* to account for demographic/obstetric characteristics (age, BMI, gestational age at diagnosis), hemodynamic severity (MAP, per 10 mmHg), neurologic symptomatology (visual disturbance), and key laboratory indices (uric acid per 50 μmol/L, albumin per 1 g/L, platelet count per 10 × 10^9^/L, and CRP per 5 mg/L). After adjustment, higher BMI (aOR 1.11, 95% CI 1.04–1.19; *p* = 0.002), higher MAP (aOR 1.82, 95% CI 1.34–2.49; *p* < 0.001), and higher uric acid (aOR 1.82, 95% CI 1.47–2.24; *p* < 0.001) remained associated with increased odds of renal impairment. In contrast, greater gestational age at diagnosis (aOR 0.90 per week, 95% CI 0.83–0.97; *p* = 0.005), higher serum albumin (aOR 0.85 per g/L, 95% CI 0.79–0.91; *p* < 0.001), and higher platelet count (aOR 0.94 per 10 × 10^9^/L, 95% CI 0.88–1.00; *p* = 0.043) remained associated with lower odds of renal impairment. Age and visual disturbance were not statistically significant in the adjusted model (*p* = 0.255 and *p* = 0.265, respectively), and CRP was not statistically significant after adjustment (*p* = 0.068; Table 4).

**Table 4:** Multivariable logistic regression identifying independent factors associated with renal impairment in preeclampsia.

Independent factor	Adjusted OR (95% CI)	*p* value
Age (per 1 year)	1.03 (0.98–1.09)	0.255
BMI (per 1 kg/m^2^)	1.11 (1.04–1.19)	0.002*
Gestational age at diagnosis (per 1 week)	0.90 (0.83–0.97)	0.005*
MAP (per 10 mmHg)	1.82 (1.34–2.49)	<0.001*
Visual disturbance (yes vs. no)	1.54 (0.72–3.32)	0.265
Uric acid (per 50 μmol/L)	1.82 (1.47–2.24)	<0.001*
Albumin (per 1 g/L)	0.85 (0.79–0.91)	<0.001*
Platelet count (per 10 × 10^9^/L)	0.94 (0.88–1.00)	0.043*
CRP (per 5 mg/L)	1.14 (0.99–1.31)	0.068

*p < 0.05. aOR, adjusted odds ratio; BMI, body mass index; CI, confidence interval; CRP, C-reactive protein; MAP, mean arterial pressure.

Model performance metrics indicated acceptable calibration (Hosmer–Lemeshow *χ*^2^ = 7.06, df = 8; *p* = 0.531) and good discrimination (AUC 0.858, 95% CI 0.815–0.898). Collinearity diagnostics suggested minimal multicollinearity among covariates (maximum VIF = 1.04), supporting the stability of the estimated regression coefficients (Supplementary Table S1).

In the expanded clinically adjusted model, early-onset PE was entered in place of continuous gestational age at diagnosis to avoid collinearity. After adjustment for age, BMI, early-onset PE, MAP, visual disturbance, 24-h urinary protein, serum uric acid-to-creatinine ratio, albumin, platelet count, and CRP, early-onset PE remained independently associated with renal impairment (aOR 1.68, 95% CI 1.02–2.78; *p* = 0.043). Higher BMI, higher MAP, and greater 24-h urinary protein were also associated with increased odds of renal impairment, whereas a higher serum uric acid-to-creatinine ratio and higher albumin were inversely associated with renal impairment. Age, visual disturbance, platelet count, and CRP were not statistically significant in this expanded model (Supplementary Table S2).

### 3.6 Association between renal impairment and maternal outcomes

During hospitalization, the renal-impairment group had a higher incidence of the composite adverse maternal outcome compared with controls (67.2% vs. 34.1%, *p* < 0.001). Several individual complications were also more frequent among patients with renal impairment, including ICU admission (30.5% vs. 8.8%), eclampsia (11.7% vs. 2.0%), HELLP syndrome (21.1% vs. 7.8%), DIC (8.6% vs. 1.5%), acute heart failure/pulmonary edema (14.1% vs. 3.4%), and placental abruption (14.1% vs. 4.9%) (all *p* ≤ 0.003), whereas postpartum hemorrhage did not differ significantly between groups (*p* = 0.133; Table 5). After adjustment for key clinical and laboratory covariates, renal impairment remained associated with higher odds of the composite adverse maternal outcome (adjusted OR 2.08, 95% CI 1.20–3.59; *p* = 0.009), ICU admission (adjusted OR 3.39, 95% CI 1.65–6.95; *p* < 0.001), and eclampsia (adjusted OR 3.92, 95% CI 1.09–14.14; *p* = 0.037), while the adjusted associations with HELLP syndrome and postpartum hemorrhage were not statistically significant (Supplementary Table S3).

**Table 5:** Maternal adverse outcomes during hospitalization by renal impairment status.

Maternal outcome	Renal impairment (*n* = 128)	Control (*n* = 205)	Test statistic	*p* value
Composite adverse maternal outcome†	86 (67.2)	70 (34.1)	*χ*^2^ = 34.55	<0.001*
ICU admission	39 (30.5)	18 (8.8)	*χ*^2^ = 26.13	<0.001*
Eclampsia	15 (11.7)	4 (2.0)	*χ*^2^ = 13.97	<0.001*
HELLP syndrome	27 (21.1)	16 (7.8)	*χ*^2^ = 12.37	<0.001*
Postpartum hemorrhage	21 (16.4)	22 (10.7)	*χ*^2^ = 2.26	0.133
DIC	11 (8.6)	3 (1.5)	*χ*^2^ = 9.95	0.002*
Acute heart failure/pulmonary edema	18 (14.1)	7 (3.4)	*χ*^2^ = 12.87	<0.001*
Placental abruption	18 (14.1)	10 (4.9)	*χ*^2^ = 8.63	0.003*

Data are presented as *n* (%). †Composite adverse maternal outcome was defined as the occurrence of any of the following during hospitalization: ICU admission, eclampsia, HELLP syndrome, postpartum hemorrhage, DIC, acute heart failure/pulmonary edema, or placental abruption. *p < 0.05. DIC, disseminated intravascular coagulation; HELLP, hemolysis, elevated liver enzymes, and low platelet count; ICU, intensive care unit.

### 3.7 Association between maternal renal impairment and neonatal outcomes

Neonates born to mothers with renal impairment had a lower gestational age at delivery (35.25 ± 2.40 vs. 36.51 ± 2.26 weeks, *p* < 0.001) and lower birth weight (2,351 ± 552 vs. 2,715 ± 539 g, *p* < 0.001) than those in the control group. The renal-impairment group also showed higher rates of preterm birth (78.9% vs. 62.4%, *p* = 0.002), low birth weight (60.2% vs. 32.7%, *p* < 0.001), low 5-min Apgar score (≤7; 31.2% vs. 14.6%, *p* < 0.001), and NICU admission (47.7% vs. 22.0%, *p* < 0.001; Table 6). After adjustment for key maternal covariates, renal impairment remained associated with increased odds of preterm birth (adjusted OR 2.24, 95% CI 1.22–4.14; *p* = 0.010), low birth weight (adjusted OR 2.70, 95% CI 1.56–4.68; *p* < 0.001), low 5-min Apgar score (adjusted OR 3.25, 95% CI 1.68–6.28; *p* < 0.001), and NICU admission (adjusted OR 3.28, 95% CI 1.83–5.87; *p* < 0.001; Supplementary Table S4).

**Table 6:** Neonatal outcomes during hospitalization by maternal renal impairment status.

Neonatal outcome	Renal impairment (*n* = 128)	Control (*n* = 205)	Test statistic	*p* value
Gestational age at delivery, weeks	35.25 ± 2.40	36.51 ± 2.26	*t* = −4.73	<0.001*
Birth weight, g	2,351 ± 552	2,715 ± 539	*t* = −5.90	<0.001*
Preterm birth (<37 weeks), *n* (%)	101 (78.9)	128 (62.4)	*χ*^2^ = 9.95	0.002*
Low birth weight (<2,500 g), *n* (%)	77 (60.2)	67 (32.7)	*χ*^2^ = 24.23	<0.001*
Low 5-min Apgar score (≤7), *n* (%)	40 (31.2)	30 (14.6)	*χ*^2^ = 13.10	<0.001*
NICU admission, *n* (%)	61 (47.7)	45 (22.0)	*χ*^2^ = 23.99	<0.001*

Data are presented as mean ± SD or n (%) as appropriate. *p < 0.05. NICU, neonatal intensive care unit; SD, standard deviation.

### 3.8 Sensitivity and subgroup analyses

Using an expanded renal-impairment definition incorporating KDIGO-AKI criteria increased the proportion of patients classified as having renal impairment (44.1%) compared with an SCr-only definition (37.5%), with high concordance between definitions (*κ* = 0.852). In sensitivity models adjusted for key maternal covariates, renal impairment remained associated with higher odds of adverse outcomes under both definitions. Specifically, renal impairment was consistently associated with the composite adverse maternal outcome (SCr-only: aOR 2.10, 95% CI 1.20–3.68; SCr/KDIGO: aOR 2.34, 95% CI 1.39–3.92), ICU admission (aORs 3.06–3.52), and NICU admission (aORs 2.79–3.11), suggesting the robustness of the primary associations to alternative renal-impairment definitions (Supplementary Table S5).

To further address the clinical heterogeneity between early-onset and late-onset PE, renal involvement was compared according to PE onset type. Renal impairment was more frequent in early-onset PE than in late-onset PE [96/222 (43.2%) vs. 32/111 (28.8%), *p* = 0.011]. Patients with early-onset PE also showed higher SCr, BUN, uric acid, UPCR, and 24-h urinary protein levels, as well as lower eGFR, compared with those with late-onset PE. The serum uric acid-to-creatinine ratio was lower in early-onset PE, reflecting the higher creatinine burden in this subgroup despite higher absolute uric acid levels (Supplementary Table S6).

In stratified multivariable models, the factors associated with renal impairment were generally consistent between early-onset and late-onset PE. In early-onset PE, higher BMI, higher MAP, greater 24-h urinary protein, lower serum uric acid-to-creatinine ratio, and lower albumin were independently associated with renal impairment. In late-onset PE, higher MAP, greater 24-h urinary protein, lower serum uric acid-to-creatinine ratio, and lower albumin remained associated with renal impairment, whereas BMI was no longer statistically significant, likely reflecting the smaller sample size and fewer renal-impairment events in this subgroup (Supplementary Table S7).

In stratified analyses based on gestational age at diagnosis, the association between renal impairment and the composite adverse maternal outcome was observed in early-onset PE, whereas the estimate was attenuated and not statistically significant in late-onset PE; however, formal interaction testing did not indicate significant effect modification by gestational-age stratum (p for interaction = 0.150). Similar patterns were observed across strata defined by magnesium sulfate exposure and antihypertensive therapy, with no statistically significant interactions. In the AKI severity analysis, composite adverse maternal outcome rates increased across KDIGO proxy stages, from 34.9% in patients without AKI to 59.4% in AKI stage 1 and 67.4% in AKI stage 2 (*χ*^2^ = 24.77, *p* < 0.001; trend OR per 1-stage increase, 2.15; 95% CI, 1.56–2.96; *p* < 0.001; Supplementary Table S8).

## 4 Discussion

This retrospective cohort study showed that renal impairment in preeclampsia was associated with a distinct clinical and laboratory profile at diagnosis and with higher odds of adverse maternal and neonatal outcomes during hospitalization. Patients with renal impairment were more likely to have early-onset preeclampsia, higher blood pressure levels, greater proteinuria burden, and more pronounced renal-function abnormalities. In the primary multivariable model, higher BMI, higher MAP, and higher uric acid levels were independently associated with increased odds of renal impairment, whereas later gestational age at diagnosis, higher serum albumin levels, and higher platelet counts were associated with lower odds. In the expanded clinically adjusted model incorporating PE onset type, 24-h urinary protein, and the serum uric acid-to-creatinine ratio, early-onset PE, higher MAP, greater proteinuria, lower serum uric acid-to-creatinine ratio, and lower albumin remained associated with renal impairment. These findings suggest that renal impairment in preeclampsia may reflect a combined phenotype of hemodynamic severity, placental disease severity, proteinuria burden, and systemic endothelial dysfunction rather than an isolated renal abnormality.

The association between higher MAP and renal impairment is clinically plausible because MAP reflects hemodynamic severity and overall pressure burden, both of which may contribute to renal microvascular stress and renal vulnerability in preeclampsia. Preeclampsia is a multisystem disorder characterized by endothelial dysfunction and microvascular injury, with AKI representing one of the major short-term maternal complications in severe cases (22, 23). Within this framework, MAP may capture a readily available hemodynamic component of disease severity relevant to renal involvement. Higher BMI also remained associated with renal impairment after adjustment. Elevated BMI is commonly linked to cardiometabolic risk, heightened inflammatory activity, and endothelial dysfunction, which may mark a higher-risk maternal phenotype when superimposed on placentally mediated vascular pathology. Contemporary studies of hypertensive disorders of pregnancy similarly emphasize that maternal cardiometabolic factors are associated with disease severity and end-organ involvement (24). Although causality cannot be inferred from this retrospective study, these findings support heightened clinical vigilance for renal involvement among patients presenting with elevated BMI and higher blood pressure burden.

Uric acid remained independently associated with renal impairment in the primary model. In preeclampsia, hyperuricemia may reflect reduced renal clearance, oxidative stress, and systemic endothelial dysfunction, and it is often considered together with blood pressure and proteinuria when evaluating disease severity and complication risk (25). However, because renal impairment was partly defined by serum creatinine, interpretation of uric acid-related indices requires caution. In the expanded model, greater 24-h urinary protein remained associated with renal impairment, whereas a higher serum uric acid-to-creatinine ratio was inversely associated with renal impairment. This pattern likely reflects the greater creatinine burden among patients with renal impairment despite higher absolute uric acid levels, supporting the view that uric acid should be interpreted in the context of renal clearance. Serum albumin was also inversely associated with renal impairment. Hypoalbuminemia in hypertensive disorders of pregnancy may reflect systemic capillary leak, inflammatory activation, malnutrition, and urinary protein loss, all of which may accompany severe disease and renal involvement. Current kidney health frameworks emphasize systematic assessment of renal function and proteinuria during pregnancy and the postpartum period, underscoring that biochemical indicators such as albumin may provide clinically relevant information beyond blood pressure alone (26). Similarly, platelet count was inversely associated with renal impairment in the primary model, consistent with the role of platelet consumption, endothelial activation, and microangiopathic processes in severe preeclampsia and HELLP-spectrum disease (27). Although this association was attenuated in the expanded model, platelet count remains a readily available marker that should be interpreted together with PE onset type, MAP, proteinuria burden, and biochemical indices.

Gestational age at diagnosis was inversely associated with renal impairment, and early-onset PE was more frequent among patients with renal impairment and remained significant in the expanded model. Earlier-onset disease is generally associated with more severe placental dysfunction, greater maternal complication burden, and earlier delivery. Recent reviews continue to emphasize that severe preeclampsia phenotypes are associated with AKI and other major maternal complications, as well as higher rates of prematurity and neonatal morbidity (28). In the present cohort, earlier diagnosis and early-onset PE may therefore represent a more severe clinical phenotype characterized by greater renal involvement. The stratified analyses further supported this interpretation: renal impairment was more frequent in early-onset PE, and patients with early-onset PE showed greater renal-function abnormalities and proteinuria burden than those with late-onset PE. Although the association between renal impairment and the composite adverse maternal outcome appeared more evident in early-onset PE, formal interaction testing did not show statistically significant effect modification. Therefore, the observed subgroup pattern should be interpreted as clinically informative but exploratory.

Renal impairment remained associated with higher odds of the composite adverse maternal outcome and selected severe complications, particularly ICU admission and eclampsia, after adjustment. This suggests that renal impairment may serve as a marker of higher short-term maternal risk in the acute inpatient setting. Current evidence syntheses on pregnancy-related AKI indicate that renal complications are closely associated with severe hypertensive disease phenotypes and adverse maternal outcomes, including escalation of care (29). The clustering of eclampsia, HELLP syndrome, pulmonary edema, and AKI may reflect overlapping mechanisms related to endothelial dysfunction, vasospasm, and microangiopathy. In the present study, adjusted associations with HELLP syndrome and postpartum hemorrhage were attenuated and did not reach statistical significance, possibly because of overlap between renal impairment and microangiopathic laboratory abnormalities, limited event counts for individual outcomes, and the influence of clinical management pathways such as earlier delivery, ICU transfer, or transfusion thresholds. Nevertheless, the consistent associations with ICU admission and eclampsia support the clinical relevance of renal impairment as an acute risk marker.

Neonates born to mothers with renal impairment had lower gestational age at delivery and lower birth weight, as well as higher rates of prematurity, low birth weight, low 5-min Apgar score, and NICU admission. These associations remained significant after adjustment for key maternal covariates, suggesting that maternal renal impairment may provide additional information for neonatal risk assessment. These findings are consistent with the established association between severe hypertensive disease phenotypes and adverse perinatal outcomes, which may be related to uteroplacental insufficiency, medically indicated preterm delivery for maternal indications, and fetal growth restriction (30). However, gestational age at delivery may partly mediate the association between maternal renal impairment and neonatal outcomes. Renal impairment may reflect greater maternal disease severity and contribute to medically indicated earlier delivery, which is closely related to prematurity, lower birth weight, low Apgar score, and NICU admission. Therefore, these neonatal associations should be interpreted cautiously and should not be regarded as direct causal effects of renal dysfunction on the neonate. Because gestational age at delivery may lie on the causal pathway, it was not included as an adjustment covariate in the primary neonatal outcome models to avoid overadjustment. Future prospective studies with formal mediation analysis are needed to quantify the extent to which delivery timing mediates these associations.

The primary multivariable model showed acceptable calibration, good discrimination, and minimal multicollinearity, suggesting that routinely available clinical variables may contribute to risk stratification for renal impairment. The expanded model further supported the robustness of the findings by incorporating clinically relevant dimensions of PE phenotype, proteinuria severity, and uric acid metabolism. Although the model is not intended as a definitive clinical decision tool, these findings may help inform targeted renal surveillance, including serial creatinine measurement, urine output assessment, closer hemodynamic monitoring, and early multidisciplinary coordination among obstetrics, nephrology, anesthesiology, neonatology, and critical care teams. Beyond acute hospitalization, kidney-focused guidance emphasizes systematic follow-up of renal function in women affected by hypertensive disorders of pregnancy and pregnancy-related kidney injury because of the potential for persistent impairment and subsequent chronic kidney disease risk (31). Therefore, renal impairment in preeclampsia should be considered not only an acute complication but also a marker identifying patients who may benefit from structured postpartum surveillance and counseling.

This study evaluated both factors associated with renal impairment and its short-term outcome relevance for maternal and neonatal complications within a single analytic framework, incorporating multivariable adjustment, model calibration and discrimination assessment, and collinearity diagnostics. However, several limitations should be acknowledged. First, the retrospective single-center design precludes causal inference and may limit generalizability to other clinical settings with different referral patterns and management protocols. Second, definitions of renal impairment vary across studies, and misclassification may have occurred, particularly when baseline creatinine values or complete urine output data were unavailable. Third, although clinically relevant covariates were incorporated where available, residual confounding cannot be excluded, including from unmeasured or incompletely captured factors such as antihypertensive treatment intensity, timing of magnesium sulfate administration, timing of delivery decisions, aspirin indication and adherence, and biomarkers of placental dysfunction. Fourth, the temporal relationship between some predictors and renal impairment is complex because hyperuricemia may act both as a marker of disease severity and as a consequence of reduced renal clearance. Fifth, outcomes were limited to the index hospitalization, and postpartum renal recovery, long-term maternal kidney outcomes, and longer-term neonatal outcomes were not assessed. Finally, external validation and prospective evaluation are required before risk models derived from this dataset can be considered for clinical application.

## 5 Conclusion

Renal impairment in preeclampsia was associated with higher BMI, elevated MAP, increased uric acid, earlier gestational age at diagnosis, lower serum albumin, and lower platelet count. The multivariable model showed good discrimination and acceptable calibration. Renal impairment was also associated with increased odds of adverse maternal and neonatal outcomes, including ICU admission, eclampsia, preterm birth, low birth weight, low 5-min Apgar score, and NICU admission. These findings suggest that renal impairment may serve as a clinically relevant marker for risk stratification in preeclampsia, although prospective validation is needed.

## Data Availability

The raw data supporting the conclusions of this article will be made available by the authors, without undue reservation.

## References

[1] Vera-Ponce VJ Loayza-Castro JA Ballena-Caicedo J Valladolid-Sandoval LAM Zuzunaga-Montoya FE Gutierrez De Carrillo CI. Global prevalence of preeclampsia, eclampsia, and HELLP syndrome: a systematic review and meta-analysis. Front Reprod Health. (2025) 7:1706009. doi: 10.3389/frph.2025.1706009, 41293035 PMC12640961

[2] Countouris M Mahmoud Z Cohen JB Crousillat D Hameed AB Harrington CM . Hypertension in pregnancy and postpartum: current standards and opportunities to improve care. Circulation. (2025) 151:490–507. doi: 10.1161/CIRCULATIONAHA.124.073302, 39960983 PMC11973590

[3] Lee JY Lee SH. Hypertensive disorders of pregnancy: advances in understanding and management. Clin Hypertens. (2025) 31:e1. doi: 10.5646/ch.2025.31.e1, 39944939 PMC11800281

[4] Chang KJ Seow KM Chen KH. Preeclampsia: recent advances in predicting, preventing, and managing the maternal and Fetal life-threatening condition. Int J Environ Res Public Health. (2023) 20:2994. doi: 10.3390/ijerph20042994, 36833689 PMC9962022

[5] Nelson-Piercy C Srisawat N Kashani K Lumlertgul N Murugan R Rhee H . Pregnancy-associated acute kidney injury — consensus report of the 32nd acute disease quality initiative workgroup. Nat Rev Nephrol. (2025) 21:633–46. doi: 10.1038/s41581-025-00979-6, 40681846

[6] Srialluri N Surapaneni A Chang A Mackeen AD Paglia MJ Grams ME. Preeclampsia and long-term kidney outcomes: an observational cohort study. Am J Kidney Dis. (2023) 82:698–705. doi: 10.1053/j.ajkd.2023.04.010, 37516302 PMC10818021

[7] Wang X Shields CA Ekperikpe U Amaral LM Williams JM Cornelius DC. Vascular and renal mechanisms of preeclampsia. Curr Opin Physio. (2023) 33:33. doi: 10.1016/j.cophys.2023.100655, 37009057 PMC10062189

[8] Paauw ND van der Graaf AM Bozoglan R van der Ham DP Navis G Gansevoort RT . Kidney function after a hypertensive disorder of pregnancy: a longitudinal study. Am J Kidney Dis. (2018) 71:619–26. doi: 10.1053/j.ajkd.2017.10.014, 29289477

[9] Lihme I Basit S Lihme F Damholt MB Hjorth S Nohr EA . A nationwide register-based cohort study examined the association between preeclampsia in mothers and the risk of kidney disease in their offspring. Kidney Int. (2025) 108:283–92. doi: 10.1016/j.kint.2025.04.017, 40383228

[10] Melamed N Kingdom JC Fu L Yip PM Arruda-Caycho I Hui D . Predictive and diagnostic value of the angiogenic proteins in patients with chronic kidney disease. Hypertension. (2024) 81:2251–62. doi: 10.1161/HYPERTENSIONAHA.124.23411, 39162032

[11] Pan X Peng J Chen Y Di X Li P Zhang G . SFlt-1/PlGF ratio thresholds for diagnosing pre-eclampsia in pregnant women with high blood pressure. Ultrasound Obstet Gynecol. (2025) 66:631–40. doi: 10.1002/uog.70075, 40992360 PMC12579772

[12] Thomopoulos C Hitij JB De Backer T Gkaliagkousi E Kreutz R Lopez-Sublet M . Management of hypertensive disorders in pregnancy: a position statement of the European Society of Hypertension Working Group 'hypertension in women'. J Hypertens. (2024) 42:1109–32. doi: 10.1097/hjh.0000000000003739, 38690949

[13] Dharmaraj RB Thangavel K Indirajith V George N Subramanyan G Mahendran BS . Serum albumin and uric acid levels in hypertensive patients: a cross-sectional analysis from Central Tamil Nadu, South India. Cureus. (2025) 17:e76766. doi: 10.7759/cureus.76766, 39897307 PMC11786001

[14] Ciciu E Alexandru A Cimpineanu B Musledin S Cioti C Pana C . Could low serum albumin level be an independent marker of severe preeclampsia? Healthcare (Basel). (2025) 13:1503. doi: 10.3390/healthcare13131503, 40648529 PMC12248646

[15] Zhang J Liang Y Song C Hu Z. Association between serum uric acid to albumin ratio and all-cause mortality in critically ill patients with sepsis: a retrospective study. BMC Infect Dis. (2025) 25:1718. doi: 10.1186/s12879-025-12134-4, 41398565 PMC12706968

[16] Gao LN Yan D Liu XH Chen D Guo H Liu J. Association of Systemic Inflammatory Biomarkers (NLR, MLR, PLR, SII, SIRI) with preeclampsia-related kidney injury: a retrospective observational study. J Inflamm Res. (2025) 18:12725–38. doi: 10.2147/jir.S542136, 40979495 PMC12447966

[17] Dotis J Skarlatou A Fourikou M Papadopoulou A Chochliourou E. The link between preterm birth and long-term renal consequences: current knowledge and emerging therapeutic targets. Biomedicine. (2026) 14:517. doi: 10.3390/biomedicines14030517, 41898164 PMC13024223

[18] Agrawal R Suryawanshi AG Verma K Bharti A Verma D Sharmila S. Renal challenges in pregnancy: clinical spectrum and outcomes of acute kidney injury in a tertiary care center. Cureus. (2026) 18:e104357. doi: 10.7759/cureus.104357 41918633 PMC13035186

[19] Hanna M Chock VY Kamath N Raj A Swanson JR Griffin R . Acute kidney injury and neurodevelopmental outcomes in extremely premature neonates: a secondary analysis of a randomized clinical trial. JAMA Netw Open. (2025) 8:e2543270. doi: 10.1001/jamanetworkopen.2025.43270, 41222934 PMC12612947

[20] Magee LA Brown MA Hall DR Gupte S Hennessy A Karumanchi SA . The 2021 International Society for the Study of hypertension in pregnancy classification, diagnosis & management recommendations for international practice. Pregnancy Hypertens. (2022) 27:148–69. doi: 10.1016/j.preghy.2021.09.008, 35066406

[21] ACOG. Gestational hypertension and preeclampsia: ACOG practice bulletin summary, number 222. Obstet Gynecol. (2020) 135:1492–5. doi: 10.1097/aog.0000000000003892 32443077

[22] Socol FG Bernad E Craina M Abu-Awwad SA Bernad BC Socol ID . Health impacts of pre-eclampsia: a comprehensive analysis of maternal and neonatal outcomes. Medicina Kaunas. (2024) 60:1486. doi: 10.3390/medicina60091486, 39336527 PMC11434575

[23] Kontomanolis EN Prokopakis I Koutras A Andreou E Metaxas D Boulieris G . Pregnancy-related acute kidney injury: causes and its impact on perinatal outcomes-a systematic review. J Clin Med. (2025) 14:6031. doi: 10.3390/jcm14176031, 40943787 PMC12429771

[24] Reddy M Palmer K Rolnik DL Wallace EM Mol BW Da Silva Costa F. Role of placental, fetal and maternal cardiovascular markers in predicting adverse outcome in women with suspected or confirmed pre-eclampsia. Ultrasound Obstet Gynecol. (2022) 59:596–605. doi: 10.1002/uog.24851, 34985800

[25] Zhang Y Gu X Yang N Xue Y Ma L Wang Y . Prediction models for late-onset preeclampsia: a study based on logistic regression, support vector machine, and extreme gradient boosting models. Biomedicine. (2025) 13:347. doi: 10.3390/biomedicines13020347, 40002760 PMC11853338

[26] Piccoli GB Ahmed SB Fakhouri F Garovic VD Hladunewich MA Jesudason S . Women and kidney health: conclusions from a kidney disease: improving global outcomes (KDIGO) controversies conference. Kidney Int. (2025) 108:355–79. doi: 10.1016/j.kint.2025.02.021, 40439632

[27] Ernawati E Akbar MIA Aryananda RA Bachnas MA Irwinda R Dewantiningrum J . HELLP syndrome as a major contributor to adverse maternal and neonatal outcomes among preeclamptic women: findings from a multicenter retrospective cohort study. BMC Pregnancy Childbirth. (2025) 25:1345. doi: 10.1186/s12884-025-08600-1, 41430585 PMC12750787

[28] Bernardes TP Zwertbroek EF Broekhuijsen K Koopmans C Boers K Owens M . Delivery or expectant management for prevention of adverse maternal and neonatal outcomes in hypertensive disorders of pregnancy: an individual participant data meta-analysis. Ultrasound Obstet Gynecol. (2019) 53:443–53. doi: 10.1002/uog.20224, 30697855 PMC6594064

[29] Maducolil MK Al-Obaidly S Olukade T Salama H AlQubaisi M Al Rifai H. Pre-eclampsia: incidence, determinants, and pregnancy outcomes from maternity hospitals in Qatar: a population-based case-control study. J Matern Fetal Neonatal Med. (2022) 35:7831–9. doi: 10.1080/14767058.2021.1937983, 34112060

[30] Teede HJ Bailey C Moran LJ Bahri Khomami M Enticott J Ranasinha S . Association of Antenatal Diet and Physical Activity-Based Interventions with Gestational Weight Gain and pregnancy outcomes: a systematic review and Meta-analysis. JAMA Intern Med. (2022) 182:106–14. doi: 10.1001/jamainternmed.2021.6373, 34928300 PMC8689430

[31] Davidson B Bramham K Manning K Osman A Blumenthal A Clark K . Impact of preeclampsia on long-term kidney function in a low-resourced setting. Kidney Int Rep. (2026) 11:103689. doi: 10.1016/j.ekir.2025.11.014, 41502808 PMC12769136

