# Supplementary material for: Independent factors associated with renal impairment in preeclampsia and its association with maternal and neonatal adverse outcomes
Source: Front Med (Lausanne). 2026 Jul 8;13:1832755. doi: 10.3389/fmed.2026.1832755 (PMC13388794; doi:10.3389/fmed.2026.1832755)
Supplement: Supplementary file 1 [file Table_1.DOCX]

Supplementary Table S1. Diagnostics for the Primary Multivariable Logistic Regression Model

| Metric | Value |
| --- | --- |
| Sample size, n | 333 |
| Renal impairment events, n (%) | 128 (38.4) |
| McFadden pseudo R² | 0.264 |
| Akaike information criterion (AIC) | 346.5 |
| Hosmer–Lemeshow χ² (df) | 7.06 (8) |
| Hosmer–Lemeshow P value | 0.531 |
| Discrimination (AUC, 95% CI) | 0.858 (0.815–0.898) |
| Maximum VIF | 1.04 |

These diagnostics correspond to the primary multivariable model shown in Table 4.

Variance inflation factor (VIF) by variable: Age 1.02; BMI 1.02; gestational age at diagnosis 1.02; MAP 1.02; visual disturbance 1.02; uric acid 1.04; albumin 1.04; platelet count 1.03; CRP 1.01.

Abbreviations: AIC, Akaike information criterion; AUC, area under the receiver operating characteristic curve; BMI, body mass index; CI, confidence interval; CRP, C-reactive protein; MAP, mean arterial pressure; VIF, variance inflation factor.Supplementary Table S2. Expanded Clinically Adjusted Logistic Regression Model for Renal Impairment

| Independent factor | Adjusted OR (95% CI) | P value |
| --- | --- | --- |
| Age, per 1 year | 1.03 (0.98–1.09) | 0.257 |
| BMI, per 1 kg/m² | 1.09 (1.02–1.17) | 0.008* |
| Early-onset PE, <34 weeks vs ≥34 weeks | 1.68 (1.02–2.78) | 0.043* |
| MAP, per 10 mmHg | 1.73 (1.26–2.37) | 0.001* |
| Visual disturbance, yes vs no | 1.36 (0.61–3.04) | 0.45 |
| 24-hour urinary protein, per 1 g/24 h | 1.58 (1.25–2.00) | <0.001* |
| Serum uric acid-to-creatinine ratio, per 1-unit increase | 0.36 (0.26–0.51) | <0.001* |
| Albumin, per 1 g/L | 0.87 (0.81–0.94) | <0.001* |
| Platelet count, per 10×10⁹/L | 0.95 (0.89–1.01) | 0.082 |
| CRP, per 5 mg/L | 1.09 (0.94–1.26) | 0.256 |

Data are presented as adjusted odds ratios (ORs) with 95% confidence intervals (CIs). This expanded model was constructed as a supplementary analysis to incorporate PE onset type, proteinuria severity, and serum uric acid-to-creatinine ratio. Early-onset PE was entered in place of continuous gestational age at PE diagnosis to avoid collinearity. Serum uric acid-to-creatinine ratio was calculated as serum uric acid (μmol/L) divided by serum creatinine (μmol/L).

*P < 0.05.

Abbreviations: BMI, body mass index; CI, confidence interval; CRP, C-reactive protein; MAP, mean arterial pressure; OR, odds ratio; PE, preeclampsia.

Supplementary Table S3. Adjusted Association Between Renal Impairment and Maternal Outcomes

| Outcome | Adjusted OR (95% CI) | P value |
| --- | --- | --- |
| Composite adverse maternal outcome | 2.08 (1.20–3.59) | 0.009* |
| ICU admission | 3.39 (1.65–6.95) | <0.001* |
| Eclampsia | 3.92 (1.09–14.14) | 0.037* |
| HELLP syndrome | 1.20 (0.53–2.73) | 0.660 |
| Postpartum hemorrhage | 0.81 (0.36–1.80) | 0.603 |

Adjustment strategy: Models were adjusted for BMI, gestational age at diagnosis, MAP (per 10 mmHg), uric acid (per 50 μmol/L), albumin, and platelet count (per 10×10⁹/L).

*P < 0.05.

Abbreviations: BMI, body mass index; CI, confidence interval; HELLP, hemolysis, elevated liver enzymes, and low platelet count; ICU, intensive care unit; MAP, mean arterial pressure; OR, odds ratio.

Supplementary Table S4. Adjusted Association Between Renal Impairment and Neonatal Adverse Outcomes

| Outcome | Adjusted OR (95% CI) | P value |
| --- | --- | --- |
| Preterm birth (<37 weeks) | 2.24 (1.22–4.14) | 0.010* |
| Low birth weight (<2500 g) | 2.70 (1.56–4.68) | <0.001* |
| Low 5-min Apgar score (≤7) | 3.25 (1.68–6.28) | <0.001* |
| NICU admission | 3.28 (1.83–5.87) | <0.001* |

Adjustment strategy: Models were adjusted for BMI, gestational age at preeclampsia diagnosis, MAP (per 10 mmHg), uric acid (per 50 μmol/L), and albumin.

*P < 0.05.

Abbreviations: BMI, body mass index; CI, confidence interval; MAP, mean arterial pressure; NICU, neonatal intensive care unit; OR, odds ratio.

Supplementary Table S5. Sensitivity Analysis Using Alternative Definitions of Renal Impairment

| Item / Outcome | SCr criterion only | SCr and/or KDIGO-AKI criteria | Test statistic / Effect | P value |
| --- | --- | --- | --- | --- |
| Renal impairment cases, n (%) | 125 (37.5) | 147 (44.1) | Cohen’s κ=0.852 (agreement) | — |
| Composite adverse maternal outcome | aOR 2.10 (1.20–3.68) | aOR 2.34 (1.39–3.92) | Adjusted OR (95% CI) | 0.010* / 0.001* |
| ICU admission | aOR 3.06 (1.55–6.05) | aOR 3.52 (1.86–6.66) | Adjusted OR (95% CI) | 0.001* / <0.001* |
| NICU admission | aOR 2.79 (1.66–4.70) | aOR 3.11 (1.88–5.14) | Adjusted OR (95% CI) | <0.001* / <0.001* |

Adjustment: BMI, gestational age at preeclampsia diagnosis, MAP (per 10 mmHg), uric acid (per 50 μmol/L), albumin, platelet count (per 10×10⁹/L), magnesium sulfate use, and antihypertensive therapy.

*P < 0.05.

Abbreviations: aOR, adjusted odds ratio; AKI, acute kidney injury; BMI, body mass index; CI, confidence interval; ICU, intensive care unit; KDIGO, Kidney Disease: Improving Global Outcomes; MAP, mean arterial pressure; NICU, neonatal intensive care unit; OR, odds ratio; SCr, serum creatinine.

Supplementary Table S6. Renal Involvement and Renal-Related Laboratory Findings According to PE Onset Type

| Variable | Early-onset PE (n=222) | Late-onset PE (n=111) | Test statistic | P value |
| --- | --- | --- | --- | --- |
| Renal impairment, n (%) | 96 (43.2) | 32 (28.8) | χ²=6.50 | 0.011* |
| Serum creatinine, μmol/L | 92.80 ± 27.50 | 77.20 ± 20.00 | t=5.89 | <0.001* |
| Blood urea nitrogen, mmol/L | 5.90 ± 1.65 | 5.00 ± 1.35 | t=5.31 | <0.001* |
| Uric acid, μmol/L | 395.00 ± 74.00 | 361.70 ± 63.00 | t=4.28 | <0.001* |
| Serum uric acid-to-creatinine ratio | 4.48 ± 1.15 | 5.05 ± 1.12 | t=-4.34 | <0.001* |
| eGFR, mL/min/1.73 m² | 90.60 ± 20.20 | 100.30 ± 17.80 | t=-4.48 | <0.001* |
| UPCR, g/g, median [IQR] | 0.92 [0.55–1.48] | 0.62 [0.43–0.95] | U=14922 | <0.001* |
| 24-hour urinary protein, g/24 h, median [IQR] | 1.65 [1.00–2.40] | 1.08 [0.68–1.70] | U=15084 | <0.001* |
| Urine protein dipstick, n (%)† |  |  | χ²=16.25 | 0.001* |
| Negative/trace | 22 (9.9) | 24 (21.6) |  |  |
| 1+ | 72 (32.4) | 47 (42.3) |  |  |
| 2+ | 82 (36.9) | 26 (23.4) |  |  |
| ≥3+ | 46 (20.7) | 14 (12.6) |  |  |

Data are presented as mean ± standard deviation, median [interquartile range], or n (%) as appropriate.

†The χ² statistic and P value refer to the overall comparison of urine protein dipstick distribution between early-onset and late-onset PE.

*P < 0.05.

Abbreviations: BUN, blood urea nitrogen; eGFR, estimated glomerular filtration rate; IQR, interquartile range; PE, preeclampsia; UPCR, urine protein-to-creatinine ratio.

Supplementary Table S7. Stratified Multivariable Logistic Regression Models for Renal Impairment According to PE Onset Type

| Independent factor | Early-onset PE (n=222; renal impairment events=96), aOR (95% CI) | P value | Late-onset PE (n=111; renal impairment events=32), aOR (95% CI) | P value |
| --- | --- | --- | --- | --- |
| Age, per 1 year | 1.02 (0.97–1.08) | 0.462 | 1.05 (0.97–1.14) | 0.231 |
| BMI, per 1 kg/m² | 1.10 (1.02–1.19) | 0.014* | 1.08 (0.97–1.21) | 0.157 |
| MAP, per 10 mmHg | 1.72 (1.20–2.46) | 0.003* | 1.76 (1.08–2.87) | 0.023* |
| 24-hour urinary protein, per 1 g/24 h | 1.52 (1.17–1.99) | 0.002* | 1.69 (1.07–2.66) | 0.024* |
| Serum uric acid-to-creatinine ratio, per 1-unit increase | 0.39 (0.27–0.57) | <0.001* | 0.34 (0.18–0.65) | 0.001* |
| Albumin, per 1 g/L | 0.87 (0.80–0.95) | 0.001* | 0.88 (0.78–0.99) | 0.038* |

Data are presented as adjusted odds ratios (aORs) with 95% confidence intervals (CIs). Early-onset PE was defined as PE diagnosed before 34 weeks of gestation, and late-onset PE was defined as PE diagnosed at or after 34 weeks of gestation. Separate reduced multivariable models were fitted within each PE onset stratum to avoid over-parameterization, particularly in the late-onset PE subgroup.

*P < 0.05.

Abbreviations: aOR, adjusted odds ratio; BMI, body mass index; CI, confidence interval; MAP, mean arterial pressure; PE, preeclampsia.

Supplementary Table S8. Subgroup and Severity-Stratified Analyses for Composite Adverse Maternal Outcome

| Analysis | Stratum / Category | n | Composite events, n (%) | Adjusted OR (95% CI) § | P value |
| --- | --- | --- | --- | --- | --- |
| Gestational-age strata | Early-onset PE (GA at diagnosis <34 w) | 222 | 112 (50.5) | 2.53 (1.31–4.89) | 0.006* |
|  | Late-onset PE (GA at diagnosis ≥34 w) | 111 | 44 (39.6) | 1.75 (0.67–4.58) | 0.257 |
|  | P for interaction (renal × GA stratum) | — | — | — | 0.15 |
| Treatment strata | MgSO₄: Yes | 268 | 133 (49.6) | 2.16 (1.19–3.90) | 0.011* |
|  | MgSO₄: No | 65 | 23 (35.4) | 4.16 (0.96–18.03) | 0.057 |
|  | P for interaction (renal × MgSO₄) | — | — | — | 0.675 |
|  | Antihypertensive: Yes | 287 | 144 (50.2) | 1.78 (0.98–3.24) | 0.059 |
|  | Antihypertensive: No | 46 | 12 (26.1) | 3.07 (1.10–8.52) | 0.032* |
|  | P for interaction (renal × antihypertensive) | — | — | — | 0.65 |
| Severity strata (KDIGO proxy) | No AKI (stage 0) | 186 | 65 (34.9) | Reference | — |
|  | AKI stage 1 | 101 | 60 (59.4) | —† | —† |
|  | AKI stage 2 | 46 | 31 (67.4) | —† | —† |
|  | Across-group difference | — | — | χ²=24.77 | <0.001* |
|  | Trend test (per 1-stage increase) | — | — | OR 2.15 (1.56–2.96) | <0.001* |

§ Subgroup aORs were adjusted for BMI, GA at diagnosis, MAP, uric acid, albumin, and platelet count within each stratum.

† The severity-stratified section reports the unadjusted across-group χ² test and an ordinal-stage trend model. Stage-specific adjusted ORs were not additionally estimated to avoid over-parameterization given the relatively small AKI stage 2 sample.

*P < 0.05.

Abbreviations: AKI, acute kidney injury; BMI, body mass index; CI, confidence interval; GA, gestational age; KDIGO, Kidney Disease: Improving Global Outcomes; MAP, mean arterial pressure; MgSO₄, magnesium sulfate; OR, odds ratio; PE, preeclampsia.
